# Fish, Long-Chain *n*-3 PUFA and Incidence of Elevated Blood Pressure: A Meta-Analysis of Prospective Cohort Studies

**DOI:** 10.3390/nu8010058

**Published:** 2016-01-21

**Authors:** Bo Yang, Mei-Qi Shi, Zi-Hao Li, Jian-Jun Yang, Duo Li

**Affiliations:** 1 Department of Food Science and Nutrition, Zhejiang University, Hangzhou 310058, China; ybzju@zju.edu.cn (B.Y.); 21513038@zju.edu.cn (Z.-H.L.); 2 School of Public Health, Ningxia Medical University, Yinchuan 750004, China; shimeiqi@zju.edu.cn (M.-Q.S.); yangjianjun_1970@163.com (J.-J.Y.)

**Keywords:** fish, *n*-3 PUFA, blood pressure, meta-analysis

## Abstract

Results from prospective cohort studies on fish or long-chain (LC) *n*-3 polyunsaturated fatty acid (PUFA) intake and elevated blood pressure (EBP) are inconsistent. We aimed to investigate the summary effects. Pertinent studies were identified from PubMed and EMBASE database through October 2015. Multivariate-adjusted risk ratios (RRs) for incidence of EBP in the highest verses the bottom category of baseline intake of fish or LC *n*-3 PUFA were pooled using a random-effects meta-analysis. Over the follow-up ranging from 3 to 20 years, 20,497 EBP events occurred among 56,204 adults from eight prospective cohort studies. The summary RR (SRR) was 0.96 (95% CI: 0.81, 1.14; *I*^2^ = 44.70%) for fish in four studies, and 0.73 (95% CI: 0.60, 0.89; *I*^2^ = 75.00%) for LC *n*-3 PUFA in six studies (three studies for biomarker vs. three studies for diet). Circulating LC *n*-3 PUFA as biomarker was inversely associated with incidence of EBP (SRR: 0.67; 95% CI: 0.55, 0.83), especially docosahexaenoic acid (SRR: 0.64; 95% CI: 0.45, 0.88), whereas no significant association was found for dietary intake (SRR: 0.80; 95% CI: 0.58, 1.10). The present finding suggests that increased intake of docosahexaenoic acid to improve its circulating levels may benefit primary prevention of EBP.

## 1. Introduction

Elevated blood pressure (BP) has been known to be a strong modifiable risk factor for stroke, coronary heart disease (CHD), and early mortality worldwide [1,2]. Fish consumption plays an important role in the modulation of BP in hypertensive and normotensive adults [3,4,5]. Long-chain (LC) *n*-3 polyunsaturated fatty acid (PUFA), including 20:5*n*-3 (eicosapentaenoic acid, EPA), 22:5*n*-3 (docosapentaenoic acid, DPA) and 22:6*n*-3 (docosahexaenoic acid, DHA), are mainly found in fish and other marine products. Four previous meta-analyses of clinical trials showed that fish oil or LC *n*-3 PUFA supplements can dose-dependently lower BP in hypertensive patients but not in normotensive individuals [6,7,8,9]. However, investigations using animal models have shown that diets enriched in *n*-3 PUFA can protect against induced BP elevations [10,11], and dietary deficiency of *n*-3 PUFA in young rats was associated with development of hypertension in later life [12]. Some observational studies have reported that an inverse association between fish or LC *n*-3 PUFA consumption and BP elevations in normotensive participants [13,14,15], while others found no association [16,17]. Most observational studies use dietary questionnaires to estimate intake, which is generally a poor reflection of the usual intake of an individual. Typical fish consumption in the US and Europe is relatively low and makes it difficult to identify associations. In addition, hypertensive individuals who changed their dietary habit after the diagnosis may not have been excluded from study populations, which may also bias benefit for BP towards null.

Circulating levels of 20:5*n*-3 and 22:6*n*-3 were strongly correlated with fish or fish oil consumption, whereas 22:5*n*-3 was elongated from and retroconverted to 20:5*n*-3. In contrast to dietary estimations, circulating levels of LC *n*-3 PUFA as biomarker can objectively reflect both dietary consumption and biologically relevant processes. Numerous studies on diet or biomarkers of LC *n*-3 PUFA and BP have been cross-sectional or case-control designs, rather than prospective cohort studies. The potential for dietary change secondary to the diagnosis of high BP can be minimized in prospective cohort studies, due to exclusion of individuals with known hypertension at baseline. Nevertheless, results from prospective cohort studies of fish or LC *n*-3 PUFA consumption in relation to elevated BP remain inconsistent [16,18,19,20]. Thus, whether fish or LC *n*-3 PUFA intake is associated with reduced risk of elevated BP in normotensive populations is still unclear. The aim of the present systematic review and meta-analysis was to quantitatively evaluate associations between fish or LC *n*-3 PUFA intake (diet *vs.* biomarker) and incidence of elevated BP with available data from prospective cohort studies. We hypothesized that LC *n*-3 PUFA intake is inversely associated with incidence of EBP, especially 22:6*n*-3.

## 2. Methods

### 2.1. Literature Research

Systematic literature searches were conducted to identify prospective cohort studies of fish or LC *n*-3 PUFA with risk of elevated BP from EMBASE, the Cochrane Library and PubMed up to October 2015, respectively. The full details are presented in the supplementary online-data. Our search was restricted to human studies that were published in English, and duplicated studies were excluded. Authors were not contacted for the detailed information of primary studies and unpublished studies. We searched systematic reviews from the above-mentioned database, and checked the reference lists to identify publications that might have been missed.

### 2.2. Eligibility Criteria

The relevant studies were included if they met the following inclusion criteria: (1) Participants: Adults of any age located in different countries; (2) Exposure of interest: Assessment of fish or LC *n*-3 PUFA intake, and quantitative determination of total or individual (20:5*n*-3, 22:5*n*-3 and 22:6*n*-3) in circulating blood (serum/plasma/whole blood/erythrocytes); (3) Outcomes: Evaluation of elevated BP based on a BP cutoff value (systolic BP (SBP) ≥ 130 mm Hg and (or) diastolic BP (DBP) ≥ 85 mm Hg) or hypertension (SBP ≥ 140 mm Hg and (or) DBP ≥ 90 mm Hg), and reporting multivariate-adjusted relative risk (RR) with 95% confidence intervals (CI); and (4) Study design: prospective cohort study (cohort, nested case-control, and case-cohort study).

### 2.3. Data Extraction

Data extraction was completed independently and performed twice by two investigators, and disagreements were reconciled by consensus. The following data was extracted from each publication: participant characteristics (baseline age range, gender and countries), duration of follow-up, baseline fish consumption or LC *n*-3 PUFA intake as exposure of interest, exposure measurement (dietary estimations or laboratory analyses), exposure source (diet or biomarker) and multivariate-adjusted RR with 95% CI for all categories of fish or LC *n*-3 PUFA (diet or biomarker) and multiple adjustment for potential covariates.

Odds ratios (OR) in nested case-control studies were regarded as RR directly. If eligible studies reported hazard ratio (HR) with 95% CI, each HR was assumed to approximate RR. To standardize units of fish intake, we first converted frequency into grams per day (g/day). The amount of fish consumption (g/day) was estimated by multiplying the frequency of consumption (servings per day) by the corresponding portion size (grams per serving). If a publication reported servings per day as unit of measure in fish consumption, we transferred the fish amount to grams according to descriptions of the publication. If no description of portion size was reported, we deemed it to be 105 grams per serving [21]. We also defined LC *n*-3 PUFA as the sum of 22:6*n*-3, 22:5*n*-3, and 20:5*n*-3. If a publication reported individual LC *n*-3 PUFA as interest exposure only, RR for individual LC *n*-3 PUFA can be combined to approximately represent RR for total LC *n*-3 PUFA in the publication using a fixed effect model. In addition, if the individual study only reported RR based on gender (male *vs.* female), age (middle *vs.* elderly), or ethnic classifications (white *vs.* black), the RRs for the subgroups were combined to represent a RR for the whole sample of population.

### 2.4. Statistic Analysis

Statistical analyses of the combined data were performed by STATA version 11.0 (Stata CORP, College Station, TX, USA). We firstly performed a meta-analysis for the highest verses the bottom category of baseline fish consumption, LC *n*-3 PUFA intake and biomarker, respectively. Each multivariate-adjusted RR for the highest compared with the bottom category was firstly transformed to their logarithm (logRR), and the corresponding 95% CI was used to calculate the standard error (selogRR). Summary RR (SRR) with corresponding 95% CI as the overall risk estimate for eligible prospective cohort studies was calculated by using a random-effects model described by DerSimonian and Laird [22], which considers both within-study and between-study variability. Heterogeneity across studies was evaluated with the Q test and *I*^2^ statistic [23]. An *I*^2^ value greater than 50% was regarded as indicative of heterogeneity according to Cochrane Handbook. Sensitivity analysis was performed to evaluate the possible influence of individual study on summary results. Begg’s test and Egger’s test were conducted to test the possibility of publication bias [24].

Dose-response analyses were conducted to determine a potential curvilinear (nonlinear) or linear association of fish and LC *n*-3 PUFA intake with risk of elevated BP, respectively. Individual studies with three or more categories were included in the dose-response analysis. We assigned median intake of fish or LC *n*-3 PUFA for each category as previously described [25]. Restricted cubic splines with three knots (two spline transformations) at fixed percentiles (25%, 50%, and 75%) was firstly created [26,27], and then a *P* for nonlinearity was calculated to detect potential departure from a simpler linear trend by testing the coefficient of the second spline equal to zero [28]. A linear trend was estimated to achieve the associations of each 20-g/day (first quartile ) increment of fish and each 150-mg/day (first quartile ) increment of LC *n*-3 PUFA consumption with risk of elevated BP using a generalized least-squares regression model (two-stage GLST in Stata) [27], respectively. Two-tailed *p* < 0.05 was considered statistically significant.

## 3. Results

### 3.1. Literature Search

**Figure 1:**
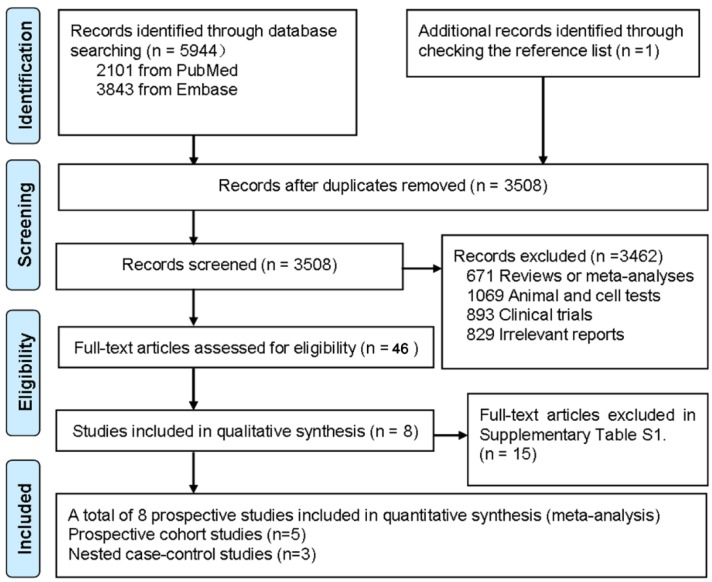
Preferred Reporting Items for Systematic Reviews and Meta-Analyses (PRISMA) Flow Diagram for included prospective cohort studies.

In total, 3508 unique citations were identified from electronic searches plus one additional article was retrieved from reference lists (Figure 1). After the titles and abstracts were screened, 23 articles were eligible for further full-text review. Eight relevant articles were available for the present meta-analysis and 15 articles were excluded for other reasons as described in Supplementary Table S1.

### 3.2. Baseline Characteristics

Overall, eight relevant prospective studies (five cohort [16,17,18,29,30] and three nested case-control studies [19,20,31]) were included in the present study (Table 1). Over the duration of follow up, which ranged from 3 to 20 years, a total of 20,497 EBP events occurred among 56,204 individuals aged 18–79 years from US (five studies) [16,17,18,19,29], Europe (one study) [31] and Asia (two studies) [20,30], respectively. Among the eight included studies, four studies evaluated EBP based on hypertension (SBP ≥ 140 mm Hg and (or) DBP ≥ 90 mm Hg) [16,18,19,29], whereas four studies evaluated EBP based on a BP cutoff value (SBP ≥ 130 mm Hg and (or) DBP ≥ 85 mm Hg) [17,20,30,31]. Both fish consumption and LC *n*-3 PUFA intake were investigated in 2 studies [18,30], fish consumption in two studies only [17,29], and dietary intake of LC *n*-3 PUFA in one study only [16]. Dietary data was collected by interviewer-administered FFQ, using servings/week (fish) and grams/day (LC *n*-3 PUFA) as unit of measure. Serum/plasma proportion of LC *n*-3 PUFA as a biomarker was determined in two studies [20,31], and erythrocytes in one study only [19]. Fatty acid (FA)composition in blood samples was quantified by gas liquid chromatography (GLC). Two studies separately included males and females [29,30], two studies only females [16,19], one study only males [31], and three studies included both males and females [17,18,20].

**Table 1:** Baseline characteristics of individual prospective cohort study.

Ref.	No. of Case/Participants	Age Range, Gender	Follow-Up Duration (Median)	Baseline Measurement		Outcomes RR (95% CI)	Multiple Adjustments
				Exposure Assessment	Exposure Range (H *vs.* L)		
[18]	999/4508	18–30 years, Both	20 years	LC *n*-3 (g/day), Fish (servings/day); FFQ	20:5*n*-3: ≥0.078 *vs.*< 0.020	0.80 (0.66–0.96)	Age, gender, ethnicity, BMI, physical activity, education, smoking, alcohol consumption, family history of hypertension, dietary intakes of total energy, sodium, and fried fish intake.
					22:6*n*-3: ≥0.096 *vs.*< 0.023	0.45 (0.37–0.55)	
					LC *n*-3: ≥0.201 *vs.* < 0.060	0.65 (0.53–0.79)	
					Fish: ≥1.258 *vs.*< 0.305	0.85 (0.70–1.03)	
[19]	516/1032	≥39 years, Female	12.9 years	Erythrocyte PL (%), GLC	20:5*n*-3: Q_4_ *vs.* Q_1_	0.69 (0.41–0.73)	Age, race, total energy intake, smoking, alcohol use, exercise, menopause status, postmenopausal hormone use, BMI, history of diabetes, and history of hypercholesterolemia.
					22:5*n*-3: Q_4_ *vs.* Q_1_	0.59 (0.34–0.90)	
					22:6*n*-3: Q_4_ *vs.* Q_1_	0.70 (0.42–1.18)	
					LC *n*-3: Q_4_ *vs.* Q_1_	0.65 (0.48–0.96)	
[20]	1000/1986	55 ± 10 years, Both	3 years	Plasma PL (%), GLC	20:5*n*-3: ≥0.76 *vs.*< 0.28	0.51 (0.33–0.80)	Age, gender, BMI, smoking, drinking, exercise, LDL cholesterol, systolic and diastolic BP, uric acid, fasting glucose levels and total fat in plasma.
					22:6n-3: ≥3.56 *vs.*<1.97	0.59 (0.38–0.92)	
[16]	13,633/28,100	≥39 years, Female	12.9 years	LC *n*-3 (g/day); FFQ	20:5*n*-3: Q_5_ *vs.* Q_1_	1.01 (0.93–1.08)	Age, race, total energy intake, drug treatment, smoking, alcohol intake, physical activity, postmenopausal status, hormone use, dietary sodium, potassium, calcium, fiber, BMI, history of diabetes, and history of hypercholesterolemia.
					22:6*n*-3: Q_5_ *vs.* Q_1_	1.07 (1.01–1.13)	
[30]	613/3504	40–69 years, Male and Female.	3.5 years	LC *n*-3 (g/day), fish (servings/week); FFQ	LC *n*-3: Q_4_ *vs.* Q_1_	0.79 (0.51–1.23)	Age, BMI, income, occupation, marital status, education level, smoking, alcohol intake, physical activity, daily intake of energy, fat, fiber, red meat, dairy products, sweetened carbonated beverages, use of multivitamin supplements, and diabetes or hypertension.
					Fish: 5–6 *vs.*<1	1.25 (0.77–2.03)	
[31]	146/880	50–70 years, Male	20 years	Serum (%), GLC	LC *n*-3: mean (SD) in noncases (1.45 (0.81))	0.74 (0.62–0.89)	BMI, smoking, and exercise.
[17]	997/4304	18–30 years, Both	15 years	Fish (times/week); dietary questionnaires	Fish: >2.5 *vs.*<0.6	1.11 (0.90, 1.38)	Age, sex, race, center, energy intake, education, physical activity, alcohol intake, smoking, and vitamin supplement.
[29]	981/5394	25–74 years, Both	10 years	Fish (times/week), FFQ	Fish: ≥1 *vs.* <1	0.84 (0.66, 1.08)	Age, smoking, history of diabetes, education, systolic BP, serum cholesterol, BMI, pulse rate, alcohol intake, and physical activity.

Ref., reference; No., number; H, the highest exposure category; L, the lowest exposure category; RR, risk ratio; LC *n*-3 PUFA, long-chain *n*-3 polyunsaturated fatty acid; g/day, gram per day; FFQ, food frequency questionnaire; 20:5*n*-3, eicosapentaenoic acid (EPA); 22:5*n*-3, docosapentaenoic acid (DPA); 22:6*n*-3, docosahexaenoic acid (DHA); BMI, body mass index; BP, blood pressure; PL, phospholipids; GLC, gas-liquid chromatography.

### 3.3. Fish Consumption and Elevated BP

**Figure 2:**
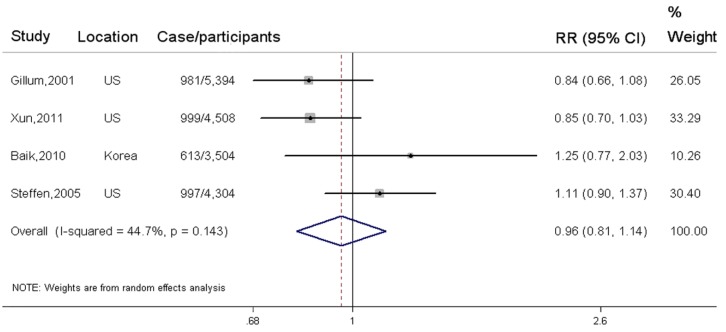
Associations between fish consumption and incidence of elevated BP in the highest verse the lowest exposure category.

In total, four independent cohort studies of fish consumption in relation to elevated BP were available for meta-analysis comparing the highest to the lowest category, with 3590 elevated BP events and 17,710 participants. Fish consumption was not significantly associated with reduced risk of elevated BP (SRR = 0.96; 95% CI: 0.81, 1.14), with a moderate heterogeneity (*I^2^* = 44.70%) (Figure 2). In addition, three studies were eligible for dose-response trend estimations. No evidence of a nonlinear association was found between fish consumption and elevated BP (*p* = 0.15 for non-linearity) (Supplementary Figure S1). Each 20-g/day increment of fish consumption was not significantly associated with reduced risk of elevated BP (pooled RR = 0.98; 95% CI: 0.94, 1.03; *p* for trend = 0.23). A sensitivity analysis which tested the influence of any individual study on the overall results suggested no significant change in pooled association estimates (Supplementary Figure S2). No possibility of publication bias was observed by visual inspection of Begg’s funnel plot (*p* for bias = 0.54) and Egger’s regression test (*p* for bias = 0.34) (Supplementary Figure S3).

All relevant cohort studies are referred to by first author, year of publication, locations, and the number of elevated BP events among participants. Squares represent study-specific risk ratio (RR), and horizontal lines represent 95% confidence interval (CI). The pooled RR estimated by a random-effect model in the highest compared with the bottom category of fish consumption is represented by the black squares. The degree of heterogeneity between individual study was indicated by I square statistic.

### 3.4. LC n-3 PUFA and Elevated BP

**Figure 3:**
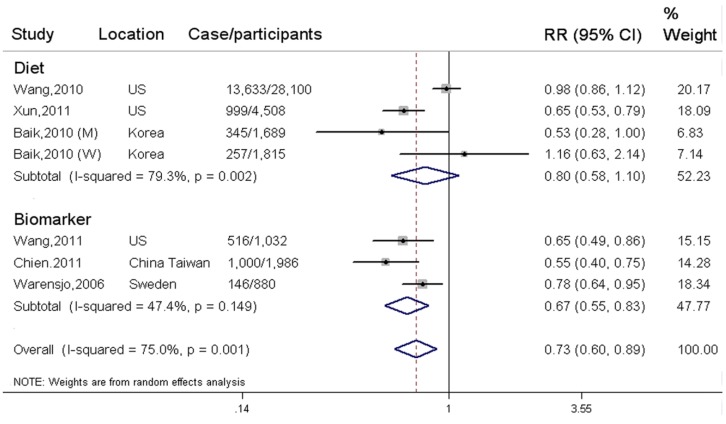
Associations between LC *n*-3 PUFA and incidence of elevated BP in the highest verse the lowest exposure category.

In total, six independent prospective cohort studies (three studies for biomarker vs. three studies for diet) were eligible to evaluate association between LC *n*-3 PUFA and incidence of elevated BP, with 16,907 elevated BP events and 38,494 participants. LC *n*-3 PUFA was inversely associated with incidence of EBP when comparing the highest with the lowest category (SRR = 0.73; 95% CI: 0.60, 0.89; *I^2^* = 75.00%) (Figure 3). The pooled association was not significantly changed in the sensitivity analysis (Supplementary Figure S4). Publication bias was not observed from Begg’s funnel plot (*p* for bias = 0.73) and Egger’s test (*p* for bias= 0.66) (Supplementary Figure S5).

Three cohort studies estimated dietary intake of LC *n*-3 PUFA, with 15,245 EBP events and 36,112 participants. The SRR was 0.80 (95% CI: 0.58, 1.10) for dietary intake of LC *n*-3 PUFA, with a high between-study heterogeneity (*I*^2^ = 79.30%). The three studies were available for trend estimation. Evidence of a nonlinear association cannot be observed between dietary intake of LC *n*-3 PUFA and incidence of EBP (*p* = 0. 57 for non-linearity) (Supplementary Figure S6). There was also no linear association between per 150 mg/day increment of LC *n*-3 PUFA intake and risk of EBP (SRR = 0.94; 95% CI: 0.84, 1.05; *p* for trend = 0.25). Two eligible studies assessed dietary intake of individual LC *n*-3 PUFA, the SRR was 0.91 (95% CI: 0.73, 1.15; *I*^2^ = 80.00%) for 20:5*n*-3, and 0.70 (95% CI: 0.30, 1.63; *I*^2^ = 98.40%) for 22:6*n*-3, respectively.

Three prospective nested case-control studies evaluated circulating levels of LC *n*-3 PUFA as biomarker, with 1662 cases and 2382 participants. Circulating LC *n*-3 PUFA was significantly associated with reduced incidence of EBP in the highest verse lowest category (SRR = 0.67; 95% CI: 0.55, 0.83; *I^2^* = 47.40%) (Figure 3). For biomarker of individual LC *n*-3 PUFA, the SRR was 0.53 (95% CI: 0.35, 0.78; *I*^2^ = 0.00%) for 20:5*n*-3 in two studies, 0.57 (95% CI: 0.36, 0.90; *I*^2^ = 0.00%) for 22:5*n*-3 in one study, and 0.64 (95% CI: 0.45, 0.89; *I*^2^ = 0.00%) for 22:6*n*-3 in two studies, respectively.

Included studies are subgrouped by dietary intake and biomarker of LC *n*-3 PUFA. All relevant cohort studies are referred to by first author, year of publication, locations, and the number of elevated BP events among participants. Squares represent study-specific risk ratio (RR); horizontal lines represent 95% confidence interval (CI); diamonds represent pooled RR from prospective cohort study. The degree of heterogeneity between individual studies was indicated by I square statistic.

## 4. Discussion

In the present meta-analysis, which included 20,497 EBP events and 56,204 individuals from eight prospective cohort studies, we cannot provide a strong evidence to support increased dietary consumption of fish or LC *n*-3 PUFA to be associated with reduced incidence of elevated BP. However, circulating LC *n*-3 PUFA as biomarker of food *n*-3 PUFA intake was significantly associated with a lower risk of elevated BP, especially 20:5n-3 and 22:6n-3, which can further build and extend on prior meta-analyses of LC *n*-3 PUFA in relation to BP.

Fish can be regarded as a package of LC *n*-3 PUFA, other nutrients and contaminants. Thus, the integrative effects of fish consumption may be reflected by the interactions between LC *n*-3 PUFA and other constituents in fish. A meta-analysis of observational studies found that fish consumption or LC *n*-3 PUFA intake was weakly associated with reduced risk of metabolic syndrome (MS) in two prospective cohort studies, but not in seven cross-sectional studies [32]. Consistent with the results from pooled analysis of seven cross-sectional studies, our findings also indicated that fish or LC *n*-3 PUFA intake was not significantly related to EBP as a component of MS. Most observational studies were primarily designed to focus on total fish rather than different species of fish or different preparation methods. Fish preparation methods may alter the relationship between fish intake and EBP by changing the lipid profile and by generating unexpected chemicals with the use of certain cooking methods. Frying fish, especially deep-frying, was found to generate oxidized lipids, considerably reduce the amount of LC *n*-3 PUFA but increase trans-fatty acids [33], which may modify the lowing-BP effects of total fish consumption [16]. Salted fish possibly added intake of salt, which could have substantially attenuated or masked a beneficial effect of fresh fish, due to high-salt intake being positively associated with BP [34]. In addition, the protective effect of fish intake might be attenuated or even reversed by other contaminants in fish, such as mercury and pesticides. Taken together, these limitations might contribute to the null association of fish intake with EBP in our meta-analysis.

The substantial BP reductions by LC *n*-3 PUFA supplementation usually occurred at relatively high doses (≥3 g/day). Nevertheless, compared with the previous evidence, our findings do not support LC *n*-3 PUFA estimated by dietary questionnaires to be associated with primary prevention of EBP. Dietary data was usually estimated by dietary questionnaires, thus underestimation of effect size may still persist due to dietary measurement errors or bias with consequent limited ability to classify dietary intake of individuals accurately. In addition, most participants included in the present meta-analysis were from US and Europe, of which typical fish intake is relatively low. Thus, the average amount of LC *n*-3 PUFA intake in study populations was insufficient to strongly affect the risk of elevated BP in initially normotensive individuals. Finally, a significant relationship could also have remained undetected if most individuals were to have an adequate fish intake. However, this perhaps happened in our study, considering that less than 18% of individuals ate no or little fish in the present study. We therefore could have missed or underestimated a pronounced association due to a small range of fish or LC *n*-3 PUFA intake.

Circulating levels of LC *n*-3 PUFA as biomarkers, compared with dietary assessment, might provide a more reliable estimation of intake. In the present study, we found that circulating LC *n*-3 PUFA was inversely associated with incidence of EBP, which can further support increased intake of fish or LC *n*-3 PUFA to be beneficial for EBP. Convincing evidence from numerous studies have indicated that circulating level of LC *n*-3 PUFA was closely correlated with increased consumption of fish, especially 20:5*n*-3 and 22:6n-3 [35,36]. Recently, a cross-sectional study suggested that plasma concentration of LC *n*-3 PUFA was positively associated with marine food intake, independent of habitual exercise, alcohol intake, and smoking habit [37]. A clinical trial compared the effects of fish (2 servings/week, 16 weeks) and fish-oil capsules (1–2 capsules/day, 16 weeks) on *n*-3 PUFA content in erythrocyte and plasma phospholipids, suggesting that consumption of equal amounts of 20:5*n*-3 and 22:6*n*-3 from fish on a weekly basis or from fish-oil capsules on a daily basis is equally effective at enriching blood lipids with *n*-3 FAs [38]. Thus, LC *n*-3 PUFA derived from fish intake rather than supplements can also be incorporated into serum/plasma, platelets, and tissue lipids to change biomembrane fluidity, increase the production of vasodilators [39], reduce cardiac adrenergic activity [40], and lower BP. The lowering-BP effects may be attributable to 22:6*n*-3 but not 20:5*n*-3, which has been supported by most previous studies. A meta-analysis of clinical studies [6] showed 22:6*n*-3 had a slightly greater dose-response effect on BP levels than 20:5*n*-3 (−1.5/−0.77 mmHg *versus* −0.93/−0.53 mmHg per gram). Mori, *et al.* found that 22:6*n*-3, but not 20:5*n*-3 supplementation, reduced the 24-h and daytime ambulatory BP in mildly hyperlipidemic men [41]. However, we did not find 22:6*n*-3 to be superior to 20:5*n*-3 with respect to EBP prevention in the present study, which may be explained by insufficient statistical power due to the small sample size. Several possible mechanisms can explain the anti-hypertensive property of 22:6*n*-3. Firstly, the 22:6*n*-3 can be more preferentially incorporated into the biomembrane than 20:5*n*-3 [42]. The incorporation of 22:6*n*-3 into cardiomyocyte membranes can inhibit the beta-adrenergic system [43], which may help to explain its anti-arrhythmic and BP-lowing effects. Furthermore, calcium/calmodulin-dependent kinase 4 (CaMK4) gene deletion can impair CaMK-mediated activation of eNOS, which induces hypertension in the mice null for CaMK4 [44]. The 22:6*n*-3 can be incorporated into endothelial membranes to stimulate ATP release from the endothelium, which leads to vasodilatation mediated by nitric oxide (NO) release [45]. The induction of NO release, together with the decrease in noradrenaline levels, is likely to be responsible for BP-lowering effect of 22:6*n*-3.

Our meta-analysis has several merits. Firstly, prospective study design minimized the possibility of selection bias, and allowed inference on temporality of associations. Secondly, the included cohort studies comprised 56,204 (men and women) with a wide age range and long-term follow-up. Thirdly, biomarker of LC *n*-3 PUFA can provide objective measures of individual *n*-3 PUFA intake, independent of dietary assessment errors and bias. Fourthly, intervention trials might be impractical for prolonged compliance to the assigned amount of fish intake, thus meta-analyses of prospective cohort studies are considered to be a powerful tool in evaluating the long-term association. Finally, food LC-PUFA is directly calculated from fish consumption in all included studies, thus the consistent results between fish and LC-PUFA perhaps strengthened our ultimate findings.

Nevertheless, the potential limitations should also be considered for this study. Firstly, our search was limited to English publications, and thus a potential bias caused by the exclusion of non-English or unpublished reports may exist; Secondly, the different exposure measurement scale across included studies were not detailed enough to allow standardization of fish consumption, thus our analysis primarily considered the highest versus the lowest exposure category; Thirdly, the diet measurement errors or misclassification in dietary estimations were likely to bias the summary results towards null. However, the use of biomarkers of dietary intake may counterbalance this point; Fourthly, in spite of comprehensive adjustments in each included study, the possibility of residual confounding caused by imprecisely measured or unmeasured factors cannot be excluded; Fifthly, the possibility of measurement bias may be inevitable, because circulating levels of *n*-3 PUFA may be subject to laboratory and biological variation during follow-up. Finally, we cannot perform a stratified analysis to determine if the pooled association estimation may be modified by strata factors, due to a limited number of studies within each subgroup.

In summary, our meta-analysis of all relevant cohort studies indicated there is no association between fish or dietary LC *n*-3 PUFA consumption and incidence of elevated BP. However, our findings show that circulating LC *n*-3 PUFA as biomarkers of dietary intake are inversely associated with incidence of EBP, especially 20:5*n*-3 and 22:6*n*-3, which suggests important public health implications for primary prevention of EBP. Stressing the consumption of food rich in LC *n*-3 PUFA to ultimately improve their circulating levels is still recommended. Nevertheless, the specific biologic mechanisms behind these conclusions remain partially unclear, and thus a replication of well-designed prospective cohort studies and further experimental work in understanding the underlying biologic mechanisms is necessary.

## Supplementary Materials

Supplementary materials are available online at www.mdpi.com/2072-6643/8/1/58/s1.
