# Supplementary material for: Fish, Long-Chain n-3 PUFA and Incidence of Elevated Blood Pressure: A Meta-Analysis of Prospective Cohort Studies
Source: Nutrients. 2016 Jan 21;8(1):58. doi: 10.3390/nu8010058 (PMC4728669; doi:10.3390/nu8010058)
Supplement: Supplementary file 1 [file nutrients-08-00058-s001.docx]

Supplementary Materials: Fish, Long-Chain *n*-3 PUFA and Incidence of Elevated Blood Pressure: A Meta-Analysis of Prospective Cohort Studies

Bo Yang, Mei-Qi Shi, Zi-Hao Li, Jian-Jun Yang and Duo Li

**Table S1.** Studies excluded after scrutiny with other reasons.

| **Reference Title** | **Reasons for Exclusion** |
| --- | --- |
| 1. A prospective study of nutritional factors and hypertension among US women | No long-chain LC *n*-3 PUFA or fish consumption in relation to elevated BP. |
| 2. Prospective study of nutritional factors, blood pressure, and hypertension among US women. | No long-chain LC *n*-3 PUFA or fish consumption in relation to elevated BP. |
| 3. The essential fatty acid status of mother and  child in pregnancy-induced hypertension: A  prospective longitudinal study. | To investigate whether the altered  essential fatty acid status observed in pregnancy-induced hypertension |
| 4. Plasma fatty acid composition and 6-year incidence of hypertension in middle-aged adults: the Atherosclerosis Risk in Communities (ARIC) Study | The odds ratio estimates incident hypertension for an interquartile increment of a fatty acid in cholesterol esters. |
| 5. Dietary protein and risk of hypertension in a Dutch older population: the Rotterdam study. | Providing the RR for dietary protein from fish in related to hypertension. |
| 6. Association between 24-h ambulatory blood pressure and erythrocyte *n*-3 polyunsaturated fatty acids in Korean subjects with hypertension | Case-control study |
| 7. Case-control study | Case-control study. |
| 8. Association between dietary patterns and metabolic syndrome in a sample of Portuguese adults | Not considering fish consumption as  interest exposure |
| 9. Association between interaction and ratio of  omega-3 and omega-6 polyunsaturated fatty acid  and the metabolic syndrome in adults | Cross-sectional study |
| 10. Mercury exposure and risk of hypertension  in US men and women in 2 prospective cohorts | No data on fish consumption as interest exposure |
| 11. Modifiable risk factors including sunlight  exposure and fish consumption are associated  with risk of hypertension in a large representative population from Macau. | Cross-sectional study |
| 12. Serum long-chain *n*-3 polyunsaturated  fatty acids, methylmercury and blood  pressure in an older population | Cross-sectional study |
| 13. Long-chain omega-3 fatty acids and blood pressure | Cross-sectional study |
| 14. Long-term fish intake is associated with better  lipid profile, arterial blood pressure, and blood  glucose levels in elderly people from Mediterranean islands (MEDIS epidemiological study | No long-chain LC *n*-3 PUFA or fish consumption in relation to elevated BP. |
| 16. Fish consumption and cardiovascular disease in the physicians’ health study: a prospective study. | Not providing the data on fish in relation to elevated BP. |


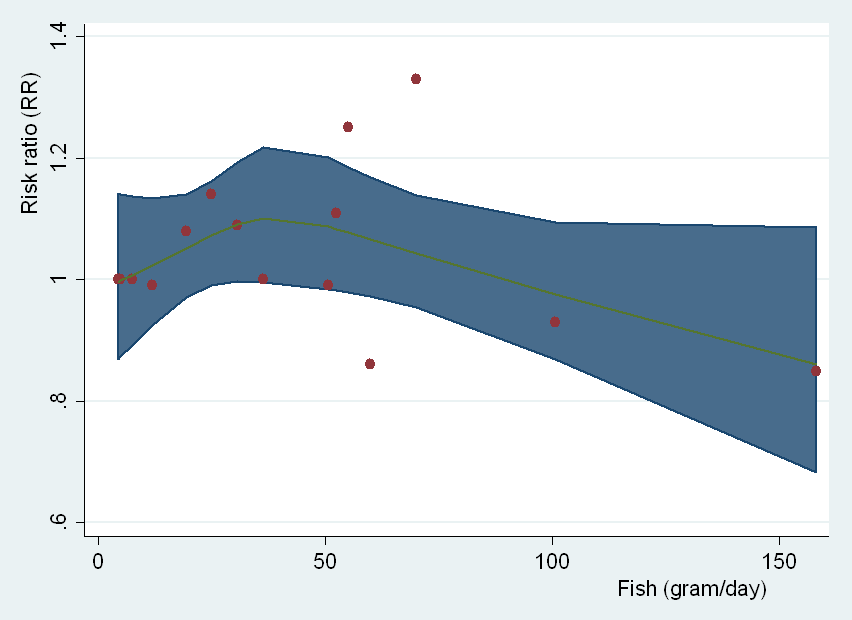


**Figure S1.** Nonlinear trend relationships between fish consumption and incidence of elevated BP using restricted cubic splines functional model.


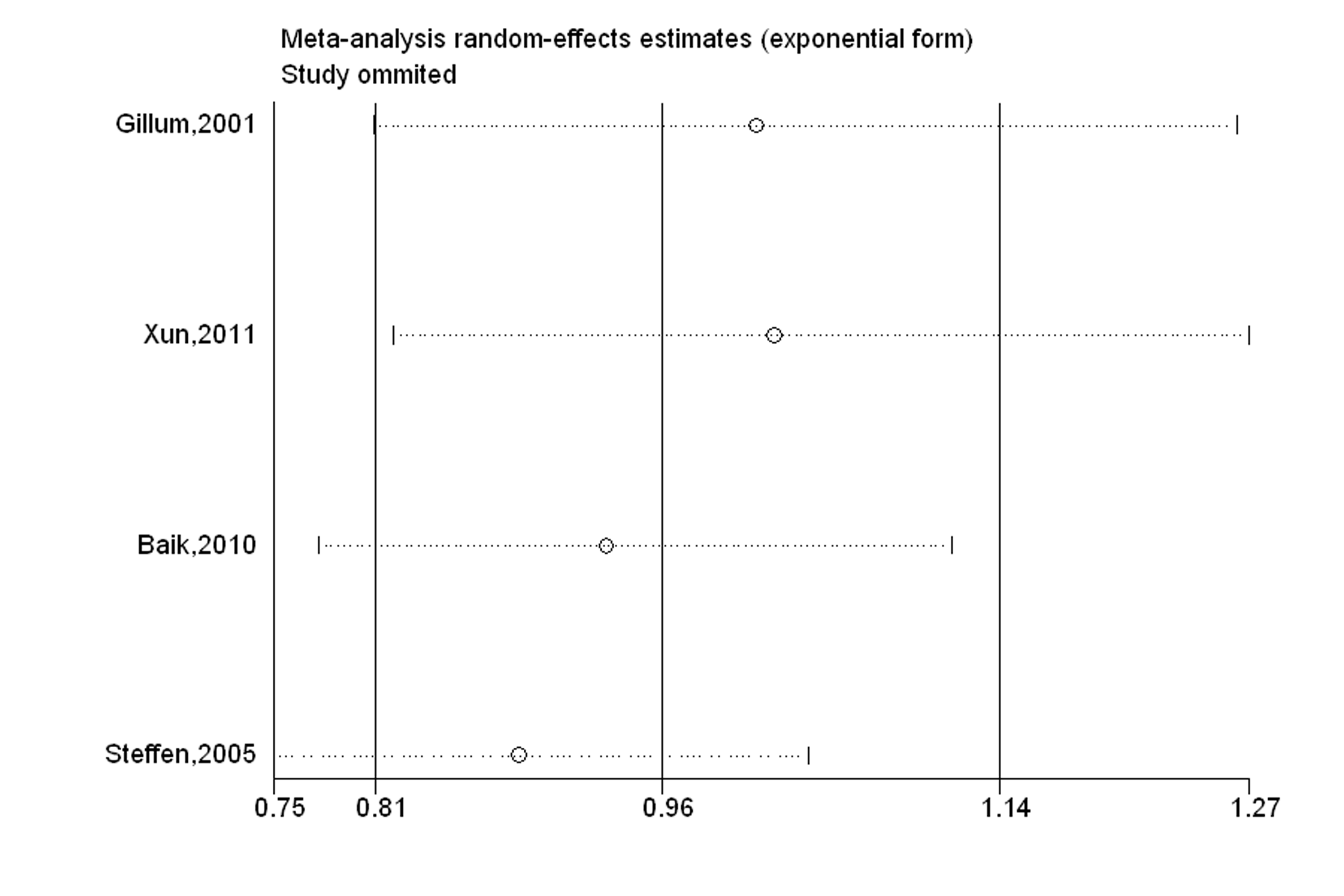


**Figure S2.** Sensitivity analysis on fish consumption with risk of elevated BP in which the pooled relative risk is re-estimated after omitting one study.


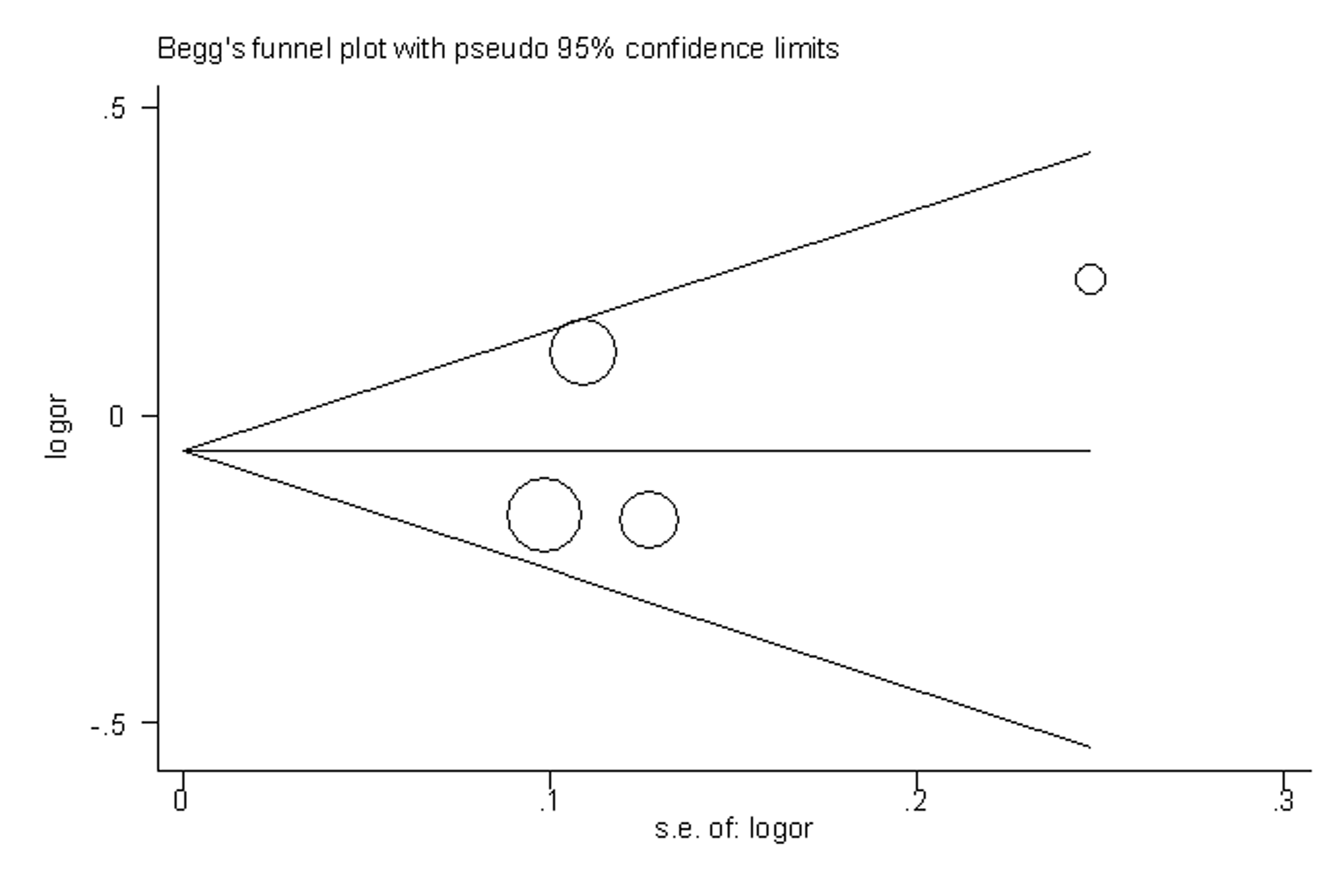


**Figure S3.** Begg’s funnel plot of incidence of elevated BP in the highest compared with the bottom category of baseline fish consumption.


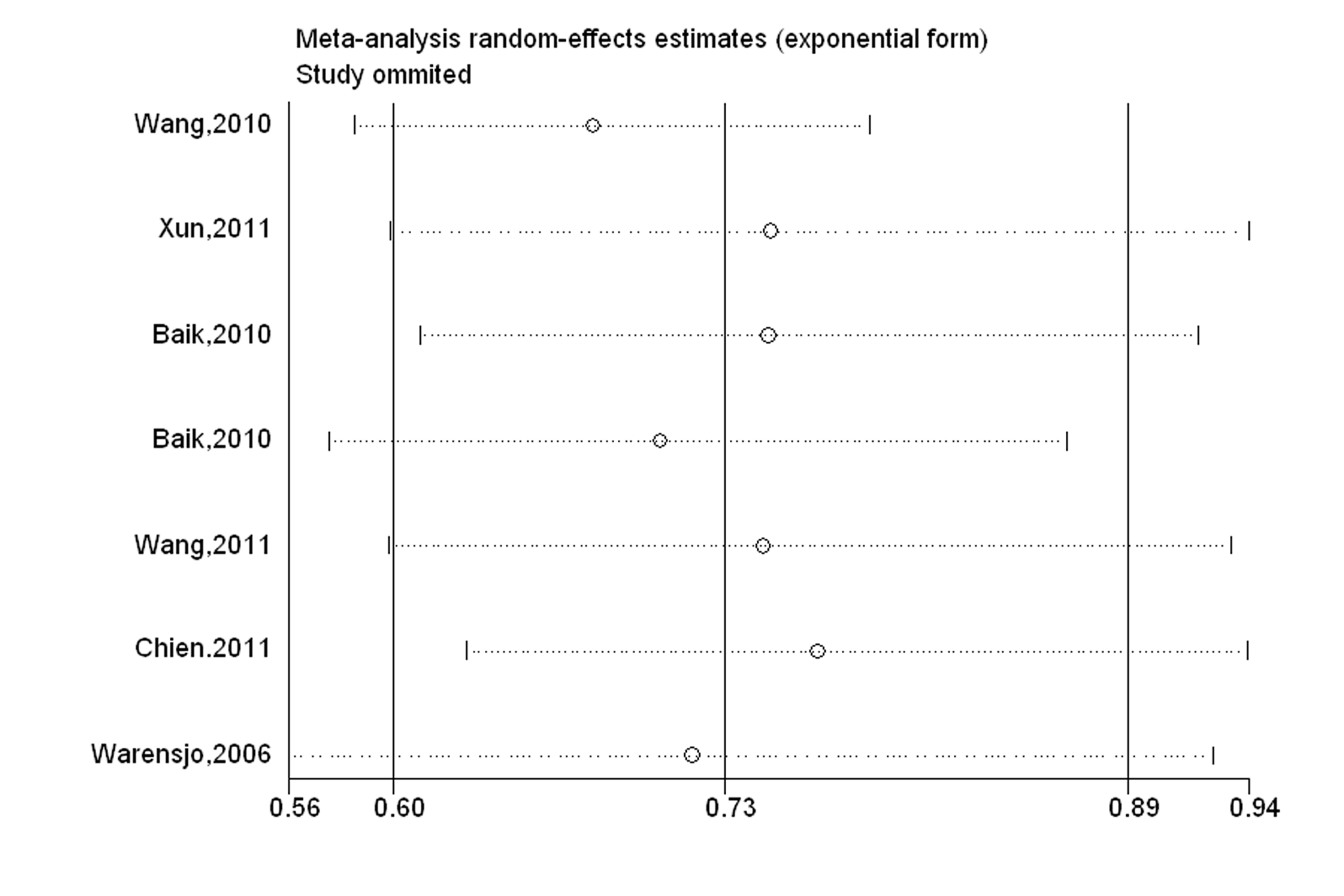


**Figure S4.** Sensitivity analysis on LC *n*-3 PUFA (diet and biomarker) with risk of elevated BP in which the pooled RR is re-estimated after omitting one study.


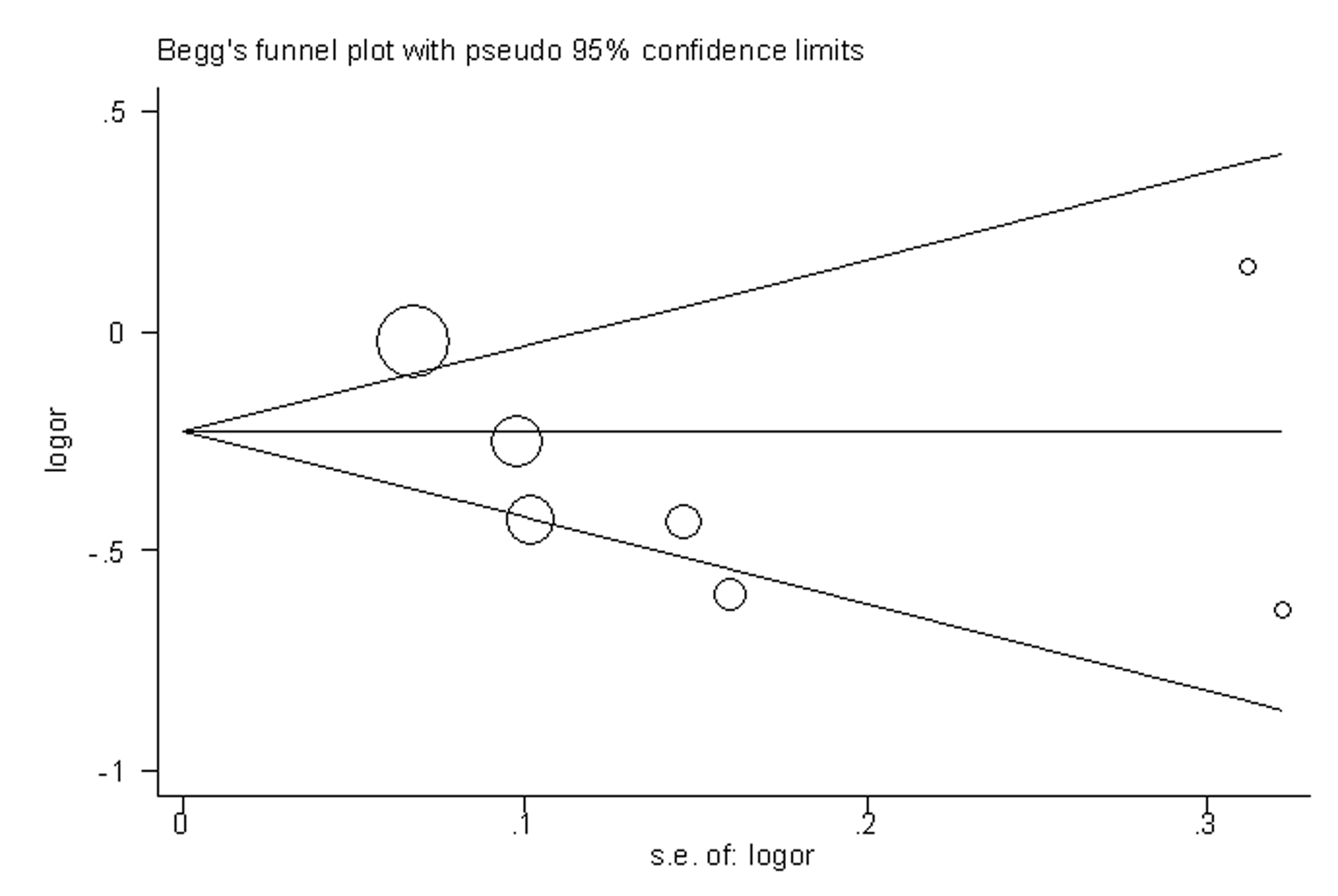


**Figure S5.** Begg’s funnel plot of incidence of elevated BP in the highest compared with the bottom category of baseline LC *n*-3 PUFA intake (diet and biomarker).


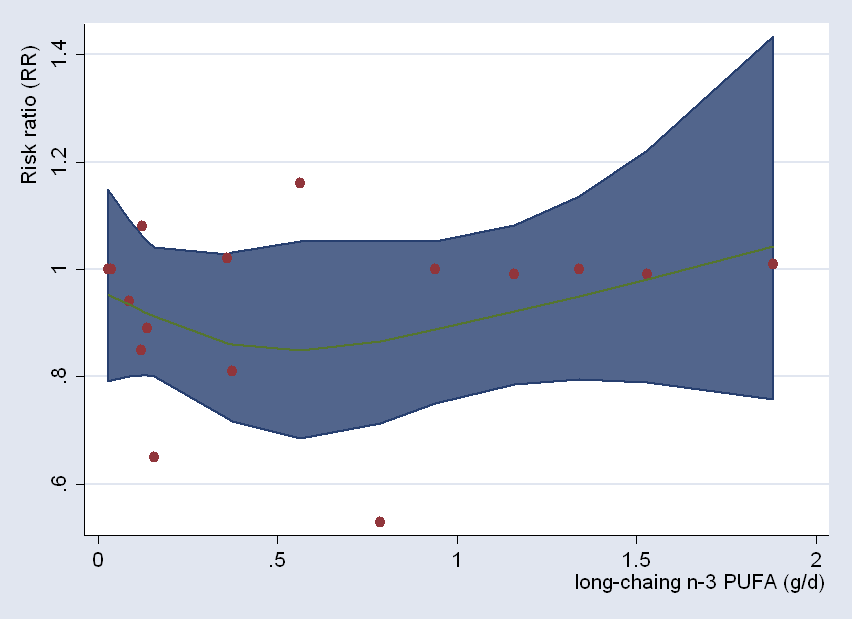


**Figure S6.** Nonlinear trend relationships between dietary intake of LC *n*-3 PUFA and incidence of elevated BP using restricted cubic splines functional model.
